# Association of obesity and insulin resistance to gestational diabetes mellitus

**DOI:** 10.6026/97320630019211

**Published:** 2023-02-28

**Authors:** Swathi Thilak, Aishwarya Rajendra, Veluri Ganesh

**Affiliations:** 1 SS Institute of Medical Sciences, Davanagere, Karnataka, India; 2 Department of Obstetrics and Gynaecology, Mavintop Hospital and IVF Center, Davangere, Karntaka - 577004, India; 3 Department of Biochemistry, Akash Institute of Medical Sciences and Research Centre, Bangalore - 562110, India

**Keywords:** Gestational diabetes mellitus, insulin resistance, HOMA-IR

## Abstract

Gestational diabetes mellitus is most commonly observed in pregnant women and it was significantly linked with dyslipidemia, obesity and insulin resistance. The present study aimed to evaluate the association of obesity, insulin resistance risk of gestational diabetes mellitus. This cross sectional study included total ninety (90) subjects out of this 30 subjects with 1st trimester, another 30 subjects were considered 2nd trimester and remaining 30 were 3rd trimester. The fasting, post parandial blood sugars, glycated haemoglobin, lipid profile and insulin levels were measured and data was recorded. There was a significantly elevated levels of fasting and post parandial blood sugars, glycated haemoglobin, total cholesterol, triacylglycerol, very low density lipoprotein, low density lipoprotein (p = 0.0001**) in all the trimesters of gestational diabetes mellitus subjects. Additionally, we also observed significantly decreased levels of high density lipoprotein, insulin and HOMA-IR (p = 0.0001**) in all the trimesters of gestational diabetes mellitus subjects. The present study IR raises as the pregnancy progresses and it is linked to bad outcomes for both the mother and the fetus. All trimesters of gestational diabetes mellitus should be screened for IR, and early treatment may assist to lessen the resulting difficulties.

##  Background:

When diabetes is discovered for the first time while pregnant, it is considered gestational diabetes (gestation). Gestational diabetes affects how the cells use sugar, similar to other types of diabetes (glucose). High blood sugar levels brought on by gestational diabetes can affect both mother and baby [1]. Typically, a number of hormones control blood sugar levels. However, as a result of altered hormone levels during pregnancy, the body has a tougher time effectively processing blood sugar [2]. This causes a spike in blood sugar. The risk factors such as being overweight or obese, not exercising regularly, pre diabetes, polycystic ovarian syndrome, family history, or having previously given birth to a child that weighed more than 9 pounds (4.1 kilograms), and specific race or ethnic groups. This leads to both hypertension, preeclampsia. a C-section is more likely to occur and this may lead to get type 2 diabetes mellitus in future [3]. In various nations, the estimated prevalence of GDM ranges from 1 to 28%. According to data from high-income nations, GDM can complicate 12.4 to 25.5% of pregnancies. According to National Guidelines and the Diabetes in Pregnancy Study Group, India, GDM is defined in India as a 2-h Oral Glucose Tolerance Test [OGTT] >140mg/dL [4]. The estimated prevalence rates for gestational diabetes in India vary greatly, ranging from 7% to about 16%. The prevalence of overweight and obesity looks to be rising in India, which may be contributing to the rising burden of gestational diabetes [5]. Numerous metabolic, biochemicals, physiological, haematological, and immunological alterations might be brought on by pregnancy. These alterations are reversible upon delivery if there are no difficulties at full term. Resistance to insulin's effects on glucose absorption and utilization has been linked to pregnancy in healthy women [6]. The ability of target organs such the liver, adipose tissue and muscle to respond to regular levels of circulating insulin is diminished in IR [7]. According to reports, pregnant women need an additional 300 kcal of energy each day in addition to their normal diet while a growing fetus's typical glucose uptake during the third trimester is around 33 mol/kg/min [8]. Due to maternal IR, the mother uses more lipids than carbohydrates for energy, sparing the fetus from consuming carbohydrates. Therefore, the mother's development of IR serves as a physiological adaptation to maintain a sufficient supply of carbohydrates for the fetus's fast growing body [9]. Growth hormone-releasing hormone (GH-RH) does not control human placental growth hormone (hPGH), a byproduct of the human growth hormone variant gene, and it is produced topically rather than pulsatile. Pituitary GH and hPGH have the same affinity for the growth hormone receptor [10]. The hPGH may also have the same diabetogenic effects as pituitary growth hormone, including hyperinsulinemia, decreased glucose uptake and glycogen synthesis triggered by insulin, and a reduction in insulin's capacity to control hepatic gluconeogenesis. Other variables, such as elevated serum cortisol levels, tumor necrosis factor (TNF), interleukin (IL)-1, and others, can disrupt the insulin signaling system during a healthy pregnancy and result in IR [11]. The IR increases during the third trimester of pregnancy and there is less writing on the first and second trimesters. The present study was designed to evaluate the association of obesity, insulin resistance risk of gestational diabetes mellitus.

## Materials and Methods:

This is cross sectional study was conducted in "District hospital, Haveri, Karnataka". A total 90 gestational diabetes mellitus subjects included in the present study and sub groped into 30 cases 1st trimester, 30 cases 2nd trimester and remaining 30 cases were 3rd trimester. All the subjects were recruited in the study after obtaining their informed consent after obtaining of ethical clearance from the institute. The study excluded any female participants who had a history of hypertension, diabetes mellitus, insulin therapy, hypoglycemic or hypolipidemic medication use, smoking, alcoholism, liver, cardiac, or renal problems, as well as any other serious illnesses. Additionally, women carrying multiple fetuses, twins, or molar pregnancies were not included in the study.

## Collection of samples:

Seven ml of overnight fasting venous blood was collected from all the subjects, and transferred, two ml into anticoagulant (sodium fluoride) tube, 2 ml into anticoagulant (EDTA) tube and 3 ml transferred into plain tube. The collected samples were separated by the process of centrifugation later transferred into properly labeled aliquots until biochemical analysis was done. The fasting and post parandial blood sugars were analysed by using glucose oxidase method, glycated haemoglobin were analysed by latex immunoturbidimetry method, lipid profile was measured by laboratory standard methods, the fasting insulin was determined by immunoassay method, Homeostatic Model Assessment of Insulin Resistance (HOMA-IR) was calculated by log { (FPG in mg/dl x FSI in µIU/ml) / 405}.

## Statistical Analysis:

The normal distribution data checked Kolmogorov Smirnov test. All the characters descriptively summarized. The mean and standard deviation about the arithmetic mean were used. The Variations between the variables was done by using analysis of variance (ANOVO) followed by posthoc analysis done in between different groups. The association between the variables was done by using Pearson Correlation analysis. The Data was compiled in Microsoft excel spread sheets and analyzed using SPSS for windows version 16.0. A p value is 0.05 was considered statistically significant.

Table 1 shows the anthropometric and biochemical parameters studied in subjects gestational diabetes mellitus. The fasting and post parandial blood sugars, HbA1c levels significantly raised in all groups of gestational diabetes mellitus subjects (p = 0.0001**). All trimesters of gestational diabetes mellitus subjects had significantly higher total cholesterol, triglycerides, very low density lipoprotein and low density lipoprotein respectively P = 0.0001**. There was a significantly decreased levels of high density lipoprotein, serum insulin and HOMA-IR concentrations was observed in all trimesters of gestational diabetes mellitus (p=0.0001**).

The association between the variables of the study by using Pearson correlation analysis is presented in Table 2. There was a significant positive correlation between the all variables respectively (p=0.0001**). Additionally, we also observed the age did not showed no significant correlation between the variables (p=0.673).

## Discussion:

Insulin resistance gradually increases throughout pregnancy as a natural adaptation to ensure that the fast developing fetus receives an adequate supply of glucose. During a typical pregnancy, glucose homeostasis is kept in check by an early adaptive increase in beta-cell glucose sensitivity and beta-cell insulin production. Hormonal, placental, genetic, and epigenetic variables, as well as an increase in visceral adipose tissue, changes in gut micro biota, and the presence of overweight or obesity concurrently, are potential pathways behind gestational insulin resistance [12]. Some beta-cell adaptation mechanisms are defective, which can significantly worsen insulin resistance and increase the risk of developing gestational diabetes mellitus (GDM). Given that glucose homeostasis quickly returns following placental ejection during delivery, the placenta unquestionably plays a crucial role in the development of prenatal insulin resistance [13]. Human chorionic gonadotropin (hCG), human placental lactogen (hPL), and human placental growth hormone (hPGH) are only a few of the pregnancy-specific hormones that the placenta secretes into the mother's bloodstream. These hormones, along with others (including prolactin), are thought to play a significant role in reprogramming maternal physiology to become insulin-resistant [14]. There is a significant increase in insulin resistance, especially in mid- and late-pregnancy, which may be related to the hormones' markedly increased production. When the concentration of plasma insulin is normal, IR refers to the state in which the action of insulin on bodily tissue is reduced. Defects in the signal transduction system, the insulin receptor's molecular structure, or the insulin receptor itself may all contribute to this [15]. In the acquired condition, the deficiency is either due to a decrease in insulin receptor affinity or an interruption in the insulin signaling cascade down the receptor. In order to counteract this IR, the beta cells in the islet of Langerhans produce more insulin, which causes hyperinsulinemia [16]. The pancreatic beta cells' capacity to produce insulin is limiting. Gradually, glucose intolerance and then diabetes mellitus develop as a result of decreased insulin production and deteriorating beta cell activity [17]. Age is one factor that affects insulin sensitivity, and several researchers have found that there is a progressive increase in IR as age increases. There was no discernible variation in the mother's age across any case groups in the current investigation (p = 0.078). The current study examines the prevalence of IR during various pregnant trimesters. The existence of IR is linked to conditions including diabetes, hypertension, and others. To avoid bias, such subjects were not included in the study. In terms of FPG concentration, there was no discernible change between any of the research groups. Additionally, controls' FSI concentrations were markedly lower than those of patients in groups I, II and III. The levels of serum insulin were significantly lower in the third trimester when compared to the second trimester and the first trimester (p 0.0001**). Researchers discovered that as the pregnancy progresses, there is a steady loss in insulin sensitivity [18]. Therefore, in response to a rise in glucose concentration, the amount of insulin generated similarly steadily increases. Normal pregnancy results in a 200% to 250% increase in insulin secretion to keep the mother's blood sugar levels stable and an approximate 50% decrease in insulin-mediated glucose elimination [19]. Gestational diabetes is more likely to develop in women with higher IR. Vicious loop of ischemia, inflammation, increased IR, dyslipidemia, and ischemia results from increased dyslipidemia which might aggravate placental ischemia [20]. It has been demonstrated in long-term studies that the majority of pregnant women who develop gestational diabetes have elevated IR brought on by changes in the insulin signaling pathway, abnormal subcellular localization of GLUT4 transporters, increased expression of the membrane glycoprotein PC-1, or diminished insulin-mediated glucose transport [21]. Premature labour, antepartum or postpartum bleeding, and foetal problems such intrauterine growth retardation or foetal overgrowth and preterm are all linked to increase IR [22]. The risk of developing metabolic syndrome, diabetes mellitus, hypertension, hyperlipidemia, and cardiovascular problems later in life is further increased by the presence of IR. All pregnant women can be advised to get screened for IR. These women's insulin sensitivity can be increased by altering their food, way of living, and level and kind of exercise. It is possible to recommend a balanced diet that includes the necessary amounts of macro and micronutrients along with a good amount of dietary fiber [23]. It should be encouraged to avoid a sedentary lifestyle and increase activity levels prior to, during, and after pregnancy. Women with elevated IR during pregnancy may benefit from gentle workouts like walking and stair climbing [24]. The timing of such intervention should be early, well before the IR-related problem manifests. Based on the study results IR significantly increased IR is risk for both mother and the fetus to develop complications.

## Conclusion:

The present study IR raises as the pregnancy progresses and it is linked to bad outcomes for both the mother and the fetus. All trimesters of gestational diabetes mellitus should be screened for IR, and early treatment may assist to lessen the resulting difficulties.

## Figures and Tables

**Table 1:** Comparison descriptive and biochemical parameters between the study subjects

**Parameter**	**Group-1**				**Group-2**		**Group- 3**			**P- Values**
Age	26.61	±	2.61	24.05	±	3.03	25.12	±	4.21	0.078++
BMI	30.62	±	1.3	32.06	±	5.69	31.33	±	2.54	0.0001**
FBS	128.31	±	8.24	136.92	±	6.85	131.29	±	5.44	0.0001**
PPBS	181.66	±	4.33	189.35	±	9.87	193.87	±	6.94	0.0001**
HbA1c	6.03	±	1.32	6.54	±	2.11	6.32	±	4.59	0.0001**
TC	263.12	±	11.05	283.55	±	19.09	235.61	±	13.65	0.0001**
TGL	182.56	±	10.22	196.79	±	22.17	191.36	±	15.21	0.0001**
HDL	32.09	±	6.12	34.54	±	9.25	38.99	±	7.62	0.0001**
VLDL	52.78	±	9.55	64.51	±	6.72	58.16	±	3.33	0.0001**
LDL	169.16	±	2.13	125.63	±	12.35	143.21	±	4.98	0.0001**
Insulin	4.16	±	0.94	3.24	±	1.33	4.97	±	2.59	0.0001**
HOMA_IR	1.97	±	0.75	1.68	±	0.97	1.39	±	0.54	0.0001**
Data expressed as mean±SD, *median (interquartile range, IQR),p value obtained using student t test or Mann Whitney U test, as appropriate, P: Probability, ++: Not Significant, *: Significant, **: Highly significant, Group 1: 1st trimester gestational diabetes subjects, Group 2: 2nd trimester gestational diabetes subjects, Group 3: 3rd trimester gestational diabetes subjects, BMI: body mass index; FBS: fasting blood sugar; PPBS: Post Parandial Blood Sugars, HbA1c: Glycated Haemoglobin, TC: total cholesterol; TGL: triglycerides; VLDL: Very Low Density Lipoprotein, LDL: Low Density Lipoprotein, HDL-C: high density lipoprotein cholesterol.

**Table 2:** Association between the variables in all the groups of gestational diabetes subjects

**Parameters**	r	**P- Values**
Age	0.542	0.673 ++
BMI	0.465	0.0001**
FBS	0.491	0.0001**
PPBS	0.364	0.0001**
HbA1c	0.198	0.0001**
TC	0.544	0.0001**
TAG	0.387	0.0001**
HDL	0.983	0.0001**
VLDL	0.579	0.0001**
LDL	0.349	0.0001**
Insulin	0.671	0.0001**
HOMA-IR	0.464	0.0001**
r: Correlation Coefficient, P: Probability, ++: Not Significant, *: Significant, **: Highly significant, BMI: body mass index; FBS: fasting blood sugar; PPBS: Post Parandial Blood Sugars, HbA1c: Glycated Haemoglobin, TC: total cholesterol; TGL: triglycerides; VLDL: Very Low Density Lipoprotein, LDL: Low Density Lipoprotein, HDL-C: high density lipoprotein cholesterol.
